# Associations between Multiple Health Indicators and Carotid Artery Intima-Media Thickness in A Healthy and Active Elderly Population

**DOI:** 10.3390/jcdd11040101

**Published:** 2024-03-28

**Authors:** Robin Pfister, Rajneesh Kaur, Gary Maesom, Ronald L. Hager

**Affiliations:** 1 Sydney Medical School, University of Sydney, City Rd, Level 2 & 3, Sydney, NSW 2050, Australia; rajneesh.kaur@sydney.edu.au; 2 Department of Nursing/Health Professions, Utah Valley University, 800 West University Parkway, Orem, UT 84058, USA; measomga@uvu.edu; 3 Department of Exercise Sciences, College of Life Sciences, Brigham Young University, Provo, UT 84602, USA; hager@byu.edu

**Keywords:** cardiovascular disease risk factors, cIMT, body mass index, blood pressure, aged, cardiorespiratory fitness

## Abstract

The purpose of this study was to examine correlations between health indicators (age, BMI, blood pressure (BP), functional strength (FS), handgrip strength, and predicted VO_2_ max) and carotid intima-media thickness (cIMT) in an active 50 years+ population. Study participants’ mean cIMT was also compared to the cIMT mean of the general population. Health screenings were conducted on 1818 participants at the Huntsman World Senior Games from 2016 to 2019. Pearson’s correlations, Spearman’s correlations, and ANOVA were performed using SPSS. Weak but significant correlations were evident between cIMT and age (r = 0.283, *p* < 0.001), systolic BP (r = 0.253, *p* = 0.001), diastolic BP (r = 0.074, *p* = 0.016), weight (r = 0.170, *p* < 0.001), height (r = 0.153, *p* < 0.001), handgrip L (r = 0.132, *p* < 0.001), handgrip R (r = 0.074, *p* < 0.029), and BMI (r = 0.07, *p* = 0.029); non-significant correlations were evident with predicted VO_2_ max (r = −0.035, *p* = 0.382), and FS (r = −0.025, *p* = 0.597). When controlling for age, systolic BP, and sex, only handgrip L (r = 0.225, *p* = 0.014) was significantly correlated with cIMT. Mean cIMT for this cohort was lower across all sexes and age-matched groups (cIMT = 0.6967 mm (±0.129)). Physical activity is linked to reduced cIMT. Most health-related indicators in this study were significantly but weakly correlated with cIMT. Additional research is needed before common indicators can be used as a surrogate for cIMT and CVD risk. Results from this study can provide clinicians with additional information to reduce CVD risk through modifiable risk factors. Classic CVD risk factors such as systolic BP and BMI should be considered in patients regardless of lifestyle.

## 1. Introduction

Atherosclerosis is the leading cause of cardiovascular disease (CVD) worldwide [1,2]. Diagnostic ultrasound can be used to assess the degree of atherosclerotic burden by measuring the thickness of the two inner layers (intima and media) of the carotid artery [3,4]. Carotid intima-media thickness (cIMT) is a marker of general artery health, and with increasing thickness there is a decrease in vascular health and a subsequent increase in cardiovascular disease risk.

The intima is the first layer within the vessel lumen, consisting of a single layer of endothelial cells. The second is the media which has multiple layers, including vascular smooth muscle, collagen, and elastic lamellae [5]. There is a natural tendency during the aging process for reduced elasticity within the vascular lumen [6]. Remodeling will occur for a variety of reasons including excess reactive oxygen species, calcification, and endothelial dysfunction [7]. Hypertension causes remodeling due to increased pulsatile pressure on the arterial wall, resulting in increased vascular smooth muscle cells (VSMCs). Hyperglycemia and elevated insulin levels also cause vascular smooth muscle proliferation and eventually increased collagen linking [8].

As a point of care option, cIMT ultrasound screening is a relatively simple, minimally invasive approach [4,9,10,11]. Because cIMT is positively associated with cardiovascular risk, ultrasound measurement of cIMT is useful for augmenting CVD risk assessment [12,13]. Nevertheless, cIMT screening may be considered expensive by some standards (average cost in the US is AUD 300.00). Therefore, patient cardiovascular risk scores are often based on classical risk factors including blood pressure, cholesterol, smoking, age, and sex [14]. There is some controversy about solely using cIMT measurements as a surrogate of cardiovascular disease risk. The 2016 European guidelines on cardiovascular disease prevention in clinical practice [15] do not suggest routine screening of patients with ultrasound cIMT measurements. They do, however, mention that ultrasound imaging may be considered in individuals with multiple conventional risk factors. A study cited in the 2016 European guidelines demonstrates a small improvement in clinical outcomes when cIMT measurements were used as a measure of cardiovascular risk in the general population ([16]). The 2016 European guidelines highlight an increased cardiovascular risk with increasing cIMT. Another cited paper demonstrated that mean cIMT is “positively and robustly associated with cardiovascular risk” [17]. Since the publication of the 2016 European guidelines, research involving very large sample sizes (*n* > 100,000) has been published, highlighting that cIMT progression may be used as a surrogate marker for cardiovascular risk [18].

Research continues to show that in the clinical setting, a comprehensive assessment of patient cardiovascular risk should be undertaken before making decision about treatment and invasive imaging options [15]. It is apparent that cIMT can play a role in the clinical setting for cardiovascular disease stratification. This study does not aim to explore the role of cIMT in clinical practice but does demonstrate that cIMT is reduced in this population sample when compared to the general United States population.

Consensus has yet to be reached on cIMT associations with some, but not all, health-related metrics such as VO_2_ max, blood pressure, strength, age, weight, and body mass index (BMI) [19,20]. Not all variance in cIMT can be explained by the risk factors studied in this paper. Metabolic risk factors such as waist circumference, fasting lipids, and fasting plasma glucose can also be used. Studies of middle-aged women after pregnancy showed that predictors of metabolic health have varying levels of association with cIMT [21]. Research on metabolic health predictors in an active healthy elderly population are limited. As already described at length in the literature, smoking causes endothelial dysfunction and cardiovascular disease progression [22]

Cardiorespiratory fitness’ association with the progression of cIMT is equivocal. Some studies performed in middle-aged individuals have reported an inverse correlation between cIMT and VO_2_ max [19,23,24]. Results from studies looking at similar younger populations have been inconclusive [25,26]. Larger population based observational trials in the 50+ population are lacking.

Cross-sectional studies have reported no association between physical strength and cIMT [26,27]. Intervention studies in younger healthy populations have shown a slight improvement of cIMT with exercise training programs [28,29]. A large prospective cohort study in adolescent males showed decreased CVD mortality in those with increased knee extension strength and handgrip strength [30]. Another study using a similar population as Ortega et al. [30] have shown inverse associations between muscular strength and cIMT [31]. Handgrip strength has routinely been used in research as a marker of physical fitness [32]. Previous studies have normalized grip strength for weight, BMI, and fat mass [33]. These studies were mostly performed in healthy populations. Our study uses it as an independent assessment, exploring its potential prognostic value without BMI and weight [34,35], as these are separate independent variables in this study. Overall, handgrip strength was used as one of two markers of physical fitness in our study. However, studies in elderly populations are surprisingly limited. One study of older men with a mean age of 81.4 (±3.2) years found an association between higher cIMT and lower handgrip strength [36], although no other association was present between cIMT and overall physical function. Studies of associations between cIMT and health markers in a population who are active and participate in frequent physical activity are limited. The apparent societal fascination for fitness and health, coupled with increased prevalence of CVD risk as the population ages, makes research centered around an active healthy population vital. A greater understanding of the aforementioned health markers associations with cIMT could lead to an increased understanding of CVD disease progression in an active aging population. Thus, this could lead to the potential for less invasive, cost-effective screenings to be part of CVD risk assessments.

The main purpose of this study is to assess correlations between health indicators (age, BMI, blood pressure (BP), functional strength (FS), handgrip strength, and predicted VO_2_ max) and cIMT in a healthy and active 50-year-old and older population. Additionally, this study makes a comparison of mean cIMT between the study participants and the general 50-year-old and older population.

## 2. Materials and Methods

The Huntsman World Senior Games (HWSG) is an annual event in St. George, UT, USA. The participants of this event are over 50 years old and older and represent more than 30 different countries; the average attendance for these games is more than 11,000 athletes annually. It is anticipated that these participants have higher activity levels and more active lifestyles than age-matched seniors in the general population.

Health screening data from 1821 participants were used (948 males, 872 females, 1 unknown). Ages ranged from 50 to 99 years old. When outliers were noted upon preparation of the data for analyses, they were excluded if values were outside of physiological limitations [*n* = 2]; for example, a diastolic BP of 650 mmHg would be considered an error in data entry and removed from the data set. Values were also not included if a person was not in a male or female category [*n* = 1]. A total of 1818 participants were included in the analysis. Total numbers for each screening outcome varied based on the number of participants who completed each screening (cIMT [*n* = 1818], BP [*n* = 1046], weight/height/BMI [*n* = 975], functional strength (FS) [*n* = 467], handgrip strength [*n* = 871], and predicted VO_2_ max [*n* = 661]).

As part of an ongoing collaboration with the HWSG, undergraduate students from local universities were involved in data collection and trained prior to the annual games event. Students were trained and subjected to practice to proficiently perform a variety of health screenings as part of a free health screening assessment for all registered HWSG participants. Screening results were shared with individual participants and recorded in an electronic database. Consent to use the data in this study was gained from the participants before the completion of screenings. Data were de-identified and collated into Excel spreadsheets. Participants were able to choose any or all of the screening tests offered. As such, not all participants who came to the health screenings participated in every screening assessment.

Intima-media thickness was measured by trained university students using a portable SonoSite ultrasound machine (Bothell, WA, USA). Longitudinal images of the far wall of the carotid artery, just proximal to the carotid bifurcation, were captured and assessed [37]. Participants had three images taken bilaterally. An average intima-media wall thickness was attained from all six images on a 10 mm longitudinal section. Images were analyzed using SonoCalc IMT 4.0 software SPSS Statistics Version 28 (SonoSite, Bothell, WA, USA). SonoCalc is an automated edge tracking software that was used to ensure accuracy and reliability of measurement [38].

Blood pressure measurements were performed with the participants in a seated position, with the blood pressure cuff on the left arm. The Omron HEM-780 (Kyoto, Japan) Automated Blood Pressure Monitor was used for all assessments. If a blood pressure assessment resulted in a high reading, blood pressure was repeated after a 5 min resting period to ensure accuracy of the assessment.

Weight was assessed using a Tanita (Tokyo, Japan) digital scale. Height was measured with a SECA 213 Portable Stadiometer (Hamburg, Germany). BMI was calculated from these two measurements using the equation BMI = kg/m^2^.

Resting predicted VO_2_ max was performed using the Polar M430 (Polar Electro Inc., Bethpage, NY, USA) heart rate wristwatch monitor and the Polar VO_2_ prediction equation [39].

Grip strength was assessed using a Jamar Handgrip Dynamometer (Los Angeles, CA, USA). Three measurements were taken on both the left and right side according to the Jamar protocol, with the highest score recorded. Participants were given as much time as required to repeat attempts.

Functional strength is a novel form of assessing core strength and stability. Multiple core exercises were used (wall sit, front plank, left and right-side plank, left and right supine bridge with leg extension, and 60-degree sit-up hold), with participants instructed to perform the tasks until failure. However, participants were given target times to classify scores into fair, good, and excellent categories with greater than 2 min being excellent. Total scores were calculated as a sum of each exercise score. A categorical variable was created from the continuous time variable according to published recommendations [40].

### Data Analysis

Statistical analysis was performed using SPSS Statistics Version 28 (Armonk, NY, USA). Based on previous published research [21,41], our sample size of *n* = 1818 (female = 872, male = 946) is sufficient to provide adequate power for multiple statistical analyses. A post hoc Bonferroni correction was applied to avoid type I errors [42].

Quartiles for cIMT, measured in millimeters, were calculated as follows: 1st <0.607, 2nd 0.607 to <0.689, 3rd 0.689 to <0.772, and 4th >0.772. One-way analysis of variance (ANOVA) was performed with cIMT divided into three categories of low (<0.599 mm, [*n* = 400]), medium (0.600–0.799 mm, [*n* = 1080]), and high (>0.800, [*n* = 338]). Quartiles of cIMT measurements were collapsed into tertiles to allow for a more straight-forward analysis and understanding. Natural cut points in the data were used. Previous research which performed similar analysis used cut points with similar cIMT measurements as this study [43].

Continuous data were checked for normality. Correlations were performed between cIMT and collated continuous variables. Pearson’s correlations were used for normally distributed variables (age, systolic BP, diastolic BP, weight, height, FF, handgrip L, handgrip R, and predicted VO_2_ max). Due to the skewed data for BMI, Spearman’s correlation was applied.

Age categories were based on decades (50–59 years, 60–69 years, 70–79 years, 80+). Age groups over 80 years were combined to maintain a meaningful group size (males *n* = 107, females *n* = 58).

## 3. Results

The cIMT variable was the dependent variable in this study. Mean cIMT across the entire population was 0.697 mm (±0.129). Mean cIMT increased for both males and females as age increased (Table 1). Mean age of the participants was 67.9 ± 8.5 years.

Significant differences in cIMT categories were noted for age, systolic BP, diastolic BP, weight, height, BMI, handgrip L, and FS. No significant difference was present for predicted VO_2_ max and handgrip R (Table 2).

Post hoc analysis indicated that there was no significant differences in predicted VO_2_ max and R hand grip strength. Despite handgrip R being significant with one-way ANOVA, (*p* = 0.032), Bonferroni correction significance was not achieved. BMI yielded significance only between the middle and high category (*p* = 0.02). All other dependent variables yielded statistically significant differences, with age and systolic BP having the highest significance *p* < 0.001 (Supplementary Table S1).

Weak significant positive correlations of cIMT were seen with age (r = 0.283, *p* < 0.001), systolic BP (r = 0.253, *p* = 0.001), diastolic BP (r = 0.074, *p* = 0.016), weight (r = 0.170, *p* < 0.001), height (r = 0.153, *p* < 0.001), handgrip L (r = 0.132, *p* < 0.001), handgrip R (r = 0.074, *p* = 0.029), and BMI (r = 0.07, *p* = 0.029). Non-significant weak negative correlations were evident in predicted VO_2_ max (r = −0.035, *p* = 0.382) and FS (r = −0.025, *p* = 0.597). (Supplementary Table S2).

Pearson’s correlations were performed while controlling for age, systolic BP, and sex (Table 3). cIMT remained positively associated with weight, BMI, handgrip L, handgrip R, diastolic BP, and height. cIMT was negatively correlated with functional strength and predicted VO_2_ max. Of these correlations, significance was found only with handgrip L.

## 4. Discussion

Associations between cIMT and multiple health indicators in athletes 50 years old and older were evaluated. Mean cIMT for this cohort was lower than the general population across both sexes and all age groups [44].

Previous studies have highlighted that there is a positive correlation between age and systolic BP with increased cIMT [45,46,47,48]. The aging process and increased pressure load in the vascular system due to hypertension will lead to adaptive hypertrophy of the arterial wall [49]. Additionally, cIMT is higher in males than females [50]. While previous research has been uncommon in an active elderly population, our data revealed a similar correlation of increased cIMT with age and systolic BP as the one detected in other populations. As such, repeats of correlations were performed while controlling for age, systolic BP, and sex.

Age- and sex-matched cIMT were lower in this study when compared to the Edinburgh Artery Study [44], which looked at an age-stratified random sample of people across 10 general medicine practices [*n* = 1106] [44]. No specific questionnaire about exercise rates was completed for this study; however, it is presumed that baseline exercise and activity is higher than average in this population due to the demographic of the participants in this study (all participants are world senior games competitors). The mean BMI of senior games athletes was 25.19 compared to the US average of 29.3 [51], and their obesity rate based on BMI (BMI > 30) was 10.73 compared to the national average for adults over 60 years old of 42.8% [52]. Additionally, mean cIMT was lower across both sexes and in all ages compared to average elderly populations [44]. The population level benefit of exercise in this study is possibly manifested in lower cIMT scores. This can be expected as individuals who exercise or are more consistent with physical activity are known to have more favorable health outcomes, as demonstrated in the studies dating as far back as 1953 [53]. Research indicates an increased life expectancy of 1.3 to 3.7 years with regular patterns of moderate to high levels of physical activity, and up to 3.3 years without cardiovascular disease [54]. Reductions in cardiovascular disease-related deaths are also correlated with decreased cIMT [54]. Some research has indicated that atherosclerosis in a healthy and active population could be reversed or stabilized with increasing age [53]. It is unlikely that many participants were following a strict plant-based diet as suggested by Esselstyn [53], and, indeed, we found no evidence of stabilizing cIMT with increased age, only attenuations in increasing thickness with age.

Importantly, while overall health outcomes of individuals in this cohort are undoubtedly better than the US national average [36,44,55,56], cIMT was still positively correlated with the expected markers of systolic BP, age, and sex. Therefore, while an individual may be considered physically fit and healthy, recommendations to maintain or improve clinical health markers such as BP and BMI should still be given. In this study, BP shows the strongest correlation with cIMT and therefore remains perhaps the most vital intervention even in healthy active individuals without apparent vascular morbidities.

Studies suggest scores on handgrip assessments may be indicative of overall strength for the whole body [57,58]. Our study found a significant but weak positive correlation between handgrip strength and cIMT (Supplementary Table S2). Results indicated that as handgrip scores increased, cIMT also increased. Even after controlling for age, systolic BP, and sex, these correlations remained for handgrip L. Handgrip R was also positively correlated but was not statistically significant (*p* = 0.069). One consideration for this might be that most of the sample was likely right-handed; therefore, handedness should be considered when using handgrip strength as a marker for cIMT.

As health-related outcomes improve, one would expect a decrease in cIMT. However, the positive correlation we found may partly be explained by the effect of increased systolic blood pressure during exercise and strenuous activity, which can lead to smooth muscle hypertrophy of the media layer of the artery. This is supported by randomized control trials [59], which highlight that exercise programs do not decrease cIMT. It is worth noting that correlations (without control variables) were significant but not considered strong (handgrip L r = 0.132, handgrip R r = 0.074). Functional strength is an indicator of core strength and stability and is a different indicator of strength than handgrip measures. While previous research conducted in general adult populations showed improved vascular status with improved skeletal muscle status [60], there were no statistically significant differences or correlations in functional strength scores across cIMT categories. After controlling for systolic BP, age, and sex, analysis showed that increased functional fitness was non-significantly (*p* = 0.161) correlated with decreased cIMT. It is important to note that mean cIMT was considerably lower in our study population, providing support for the overall effects of exercise as vital in decreasing potential atherosclerotic burden [61]. In this study, correlations were present but not significant. Future research with increased sample size may yield correlations that are statistically significant.

Some recent evidence has highlighted that the femoral artery may be superior to the carotid region [62]. However, in this study, carotid artery assessment was considered less invasive as the assessment was conducted in an open area. It should also be noted that the data collection took place prior to the publication of this paper [63]. Additional research surrounding the role of femoral artery intima-media thickness and its correlation with common health markers would be of interest.

Cardiovascular health has been linked to cardiorespiratory fitness or predicted VO_2_ max in previous research [63]. As cIMT is a predictor of systemic vascular disease, the impact of predicted VO_2_ max on cIMT has similarly been studied, however, not in a healthy and active sample of the population aged 50 years and over. Previous studies have shown that increased VO_2_ max correlated with decreased cIMT [24,64]. However, our population of healthy active adults showed no significant difference or correlations. This may be because our sample population had a relatively lower mean cIMT; therefore, a change in cIMT with respect to changes in predicted VO_2_ max in a healthy population may not be as pronounced as in the average population. After controlling for age, systolic BP, and sex, analysis showed that increasing predicted VO_2_ max led to decreased cIMT (r = −0.134); however, this correlation was not statistically significant (*p* = 0.147). Future studies with larger sample sizes should be completed to draw more robust conclusions.

## 5. Conclusions

This study reinforces that physical activity leads to decreased CVD risk, as measured by cIMT. However, even in a sample of highly active individuals, physical activity does not offset typical risk factors. Age, systolic BP, weight, and BMI were all positively correlated with greater cIMT. This suggests that these parameters may be used as surrogates for assessing vascular health and atherosclerotic cardiovascular disease. More research is needed to identify how these metrics can be applied together to form a CVD risk screening program for guiding interventions.

## Figures and Tables

**Table 1:** Mean cIMT.

	Male				Female			
Tertile	50–59 [*n* = 111]	60–69 [*n* = 356]	70–79 [*n* = 372]	80–99 [*n* = 107]	50–59 [*n* = 206]	60–69 [*n* = 371]	70–79 [*n* = 237]	80–99 [*n* = 58]
1st	0.577	0.622	0.652	0.676	0.564	0.594	0.614	0.610
2nd	0.636	0.703	0.729	0.740	0.624	0.658	0.693	0.713
3rd	0.699	0.793	0.824	0.818	0.698	0.723	0.777	0.811

**Table 2:** One-way ANOVA with Bonferroni correction for differences in age, BP, physical characteristics, predicted VO_2_ max, and strength via cIMT.

	Low (cIMT < 0.599) [*n* = 400, 22%]	Medium (cIMT 0.600–0.799) [*n* = 1080, 59.4%]	High (cIMT > 0.800) [*n* = 338, 18.6%]	*p* Value
Age	64.91 ± 8.69	67.82 ± 8.32	71.61 ± 7.63	≤0.001 ^a,b,c^
Systolic BP	126.44 ± 16.30	132.05 ± 16.55	139.89 ± 16.47	≤0.001 ^a,b,c^
Diastolic	78.50 ± 9.71	79.47 ± 9.72	81.15 ± 9.76	≤0.05 ^c^
Weigh (pounds)	159.1 ± 31.6	162.19 ± 33.60	175.77 ± 36.91	≤0.001 ^b,c^
Height (inches)	66.7 ± 3.82	67.31 ± 3.95	68.68 ± 4.08	<0.001 ^b,c^
BMI	25.02 ± 3.78	25.03 ± 4.05	26.05 ± 4.85	≤0.05 ^b,c^
Handgrip (L)	32.58 ± 10.61	35.92 ± 11.16	36.29 ± 11.35	≤0.05 ^a,c^
Handgrip (R)	33.69 ± 11.03	35.74 ± 11.27	36.42 ± 11.76	NS
VO_2_ Max	39.16 ± 8.78	39.42 ± 8.82	38.38 ± 8.14	NS
Functional Strength	13.55 ± 2.49	14.17 ± 2.15	13.36 ± 2.30	≤0.05 ^a,b^

^a^ = significant difference between low and medium. ^b^ = significant difference between medium and high. ^c^ = significant difference between low and high.

**Table 3:** Correlations while controlling for age, systolic BP, and sex.

	Dias—BP (*n* = 1045)	Weight (*n* = 975)	Height (*n* = 978)	BMI (*n* = 974)	Functional Strength (*n* = 467)	Handgrip L (*n* = 871)	Handgrip R (*n* = 871)	Predicted VO_2_ Max (*n* = 611)
r	0.007	0.161	0.088	0.126	−0.130	0.225	0.168	−0.134
p	0.940	0.081	0.346	0.175	0.161	0.014	0.069	0.175

## Data Availability

Data can be accessed upon request via direct email to the authors of this paper. Data were de-identified.

## Supplementary Materials

The following supporting information can be downloaded at: https://www.mdpi.com/article/10.3390/jcdd11040101/s1, Table S1: Multiple Comparison Bonferroni; Table S2: Correlations without Controls.

## References

[1] GBD 2016 Causes of Death Collaborators (2017). Global, regional, and national age-sex specific mortality for 264 causes of death, 1980–2016: A systematic analysis for the Global Burden of Disease Study 2016. Lancet.

[2] Lozano R., Naghavi M., Foreman K., Lim S., Shibuya K., Aboyans V., Abraham J., Adair T., Aggarwal R., Ahn S.Y. (2012). Global and regional mortality from 235 causes of death for 20 age groups in 1990 and 2010: A systematic analysis for the Global Burden of Disease Study 2010. Lancet.

[3] Wendelhag I., Gustavsson T., Suurküla M., Berglund G., Wikstrand J. (1991). Ultrasound measurement of wall thickness in the carotid artery: Fundamental principles and description of a computerized analysing system. Clin. Physiol..

[4] Hurst R.T., Ng D.W., Kendall C., Khandheria B. (2007). Clinical use of carotid intima-media thickness: Review of the literature. J. Am. Soc. Echocardiogr..

[5] Zieman S.J., Melenovsky V., Kass D.A. (2005). Mechanisms, pathophysiology, and therapy of arterial stiffness. Arterioscler. Thromb. Vasc. Biol..

[6] Van Den Munckhof I.C.L., Jones H., Hopman M.T.E., de Graaf J., Nyakayiru J., van Dijk B., Eijsvogels T.M.H., Thijssen D.H.J. (2018). Relation between age and carotid artery intima-medial thickness: A systematic review. Clin. Cardiol..

[7] Lacolley P., Regnault V., Laurent S. (2020). Mechanisms of Arterial Stiffening: From Mechanotransduction to Epigenetics. Arterioscler. Thromb. Vasc. Biol..

[8] Death A.K., Fisher E.J., McGrath K.C., Yue D.K. (2003). High glucose alters matrix metalloproteinase expression in two key vascular cells: Potential impact on atherosclerosis in diabetes. Atherosclerosis.

[9] Gazelle G.S., McMahon P.M., Siebert U., Beinfeld M.T. (2005). Cost-effectiveness analysis in the assessment of diagnostic imaging technologies. Radiology.

[10] Heiss G., Sharrett A.R., Barnes R., Chambless L.E., Szklo M., Alzola C. (1991). Carotid atherosclerosis measured by B-mode ultrasound in populations: Associations with cardiovascular risk factors in the ARIC study. Am. J. Epidemiol..

[11] Bots M.L., Hoes A.W., Koudstaal P.J., Hofman A., Grobbee D.E. (1997). Common carotid intima-media thickness and risk of stroke and myocardial infarction: The Rotterdam Study. Circulation.

[12] Stein J.H., Korcarz C.E., Hurst R.T., Lonn E., Kendall C.B., Mohler E.R., Najjar S.S., Rembold C.M., Post W.S. (2008). Use of carotid ultrasound to identify subclinical vascular disease and evaluate cardiovascular disease risk: A consensus statement from the American Society of Echocardiography Carotid Intima-Media Thickness Task Force *Endorsed by the Society for Vascular Medicine*. J. Am. Soc. Echocardiogr..

[13] Lorenz M.W., Gao L., Ziegelbauer K., Norata G.D., Empana J.P., Schmidtmann I., Lin H.-J., McLachlan S., Bokemark L., Ronkainen K. (2018). Predictive value for cardiovascular events of common carotid intima media thickness and its rate of change in individuals at high cardiovascular risk—Results from the PROG-IMT collaboration. PLoS ONE.

[14] Conroy R., Pyörälä K., Fitzgerald A., Sans S., Menotti A., De Backer G., De Bacquer D., Ducimetière D., Jousilahti P., Keil U. (2003). Estimation of ten-year risk of fatal cardiovascular disease in Europe: The SCORE project. Eur. Heart J..

[15] Piepoli M.F., Hoes A.W., Agewall S., Albus C., Brotons C., Catapano A.L., Cooney M.T., Corra U., Cosyns B., Deaton C. (2016). 2016 European Guidelines on cardiovascular disease prevention in clinical practice: The Sixth Joint Task Force of the European Society of Cardiology and Other Societies on Cardiovascular Disease Prevention in Clinical Practice (constituted by representatives of 10 societies and by invited experts) Developed with the special contribution of the European Association for Cardiovascular Prevention & Rehabilitation (EACPR). Eur. Heart J..

[16] Den Ruijter H.M., Peters S.A., Anderson T.J., Britton A.R., Dekker J.M., Eijkemans M.J., Engstrom G., Evans G.W., de Graaf J., Grobbee D.E. (2012). Common carotid intima-media thickness measurements in cardiovascular risk prediction: A meta-analysis. J. Am. Med. Assoc..

[17] Lorenz M.W., Polak J.F., Kavousi M., Mathiesen E.B., Volzke H., Tuomainen T.P., Sander D., Plichart M., Catapano A.L., Robertson C.M. (2012). Carotid intima-media thickness progression to predict cardiovascular events in the general population (the PROG-IMT collaborative project): A meta-analysis of individual participant data. Lancet.

[18] Willeit P., Tschiderer L., Allara E., Reuber K., Seekircher L., Gao L., Liao X., Lonn E., Gerstein H.C., Yusuf S. (2020). Carotid Intima-Media Thickness Progression as Surrogate Marker for Cardiovascular Risk: Meta-Analysis of 119 Clinical Trials Involving 100,667 Patients. Circulation.

[19] Kang S.J., Ko K.J. (2019). Association between resting heart rate, VO_2_ max and carotid intima-media thickness in middle-aged men. Int. J. Cardiology. Heart Vasc..

[20] Assmann G., Cullen P., Schulte H. (2002). Simple Scoring Scheme for Calculating the Risk of Acute Coronary Events Based on the 10-Year Follow-Up of the Prospective Cardiovascular Münster (PROCAM) Study. Circulation.

[21] Magri C.J., Xuereb S., Xuereb R.A., Fava S. (2023). Metabolic Health and Carotid Intima-Media Thickness: Association of Different Definitions in Women. Am. J. Cardiol..

[22] Messner B., Bernhard D. (2014). Smoking and cardiovascular disease: Mechanisms of endothelial dysfunction and early atherogenesis. Arterioscler. Thromb. Vasc. Biol..

[23] Rauramaa R., Rankinen T., Tuomainen P., Väisänen S., Mercuri M. (1995). Inverse relationship between cardiorespiratory fitness and carotid atherosclerosis. Atherosclerosis.

[24] Lakka T.A., Laukkanen J.A., Rauramaa R., Salonen R., Lakka H.M., Kaplan G.A., Salonen J.T. (2001). Cardiorespiratory fitness and the progression of carotid atherosclerosis in middle-aged men. Ann. Intern. Med..

[25] Ferreira I., Twisk J.W., Stehouwer C.D., van Mechelen W., Kemper H.C. (2003). Longitudinal changes in VO_2max_: Associations with carotid IMT and arterial stiffness. Med. Sci. Sports Exerc..

[26] Stensland-Bugge E., Bønaa K.H., Joakimsen O., Njølstad I. (2000). Sex differences in the relationship of risk factors to subclinical carotid atherosclerosis measured 15 years later: The Tromsø study. Stroke.

[27] Folsom A.R., Eckfeldt J.H., Weitzman S., Ma J., Chambless L.E., Barnes R.W., Cram K.B., Hutchinson R.G. (1994). Relation of carotid artery wall thickness to diabetes mellitus, fasting glucose and insulin, body size, and physical activity. Atherosclerosis Risk in Communities (ARIC) Study Investigators. Stroke.

[28] Moreau K.L., Silver A.E., Dinenno F.A., Seals D.R. (2006). Habitual aerobic exercise is associated with smaller femoral artery intima-media thickness with age in healthy men and women. Eur. J. Cardiovasc. Prev. Rehabil..

[29] Dinenno F.A., Tanaka H., Monahan K.D., Clevenger C.M., Eskurza I., DeSouza C.A., Seals D.R. (2001). Regular endurance exercise induces expansive arterial remodelling in the trained limbs of healthy men. J. Physiol..

[30] Ortega F.B., Silventoinen K., Tynelius P., Rasmussen F. (2012). Muscular strength in male adolescents and premature death: Cohort study of one million participants. Br. Med. J..

[31] Karabinus J.A., DeBlois J.P., Keller A., Glasgow A.C., Barreira T.V., Heffernan K.S. (2021). The Inverse Association of Muscular Strength with Carotid Intima-media and Extra-media Thickness in Women. Int. J. Sports Med..

[32] Vaishya R., Misra A., Vaish A., Ursino N., D’Ambrosi R. (2024). Hand grip strength as a proposed new vital sign of health: A narrative review of evidences. J. Health Popul. Nutr..

[33] Sayer A.A., Syddall H.E., Dennison E.M., Martin H.J., Phillips D.I., Cooper C., Byrne C.D., Hertfordshire C. (2007). Grip strength and the metabolic syndrome: Findings from the Hertfordshire Cohort Study. QJM.

[34] Leong D.P., Teo K.K., Rangarajan S., Lopez-Jaramillo P., Avezum A., Orlandini A., Seron P., Ahmed S.H., Rosengren A., Kelishadi R. (2015). Prognostic value of grip strength: Findings from the Prospective Urban Rural Epidemiology (PURE) study. Lancet.

[35] Nambi V., Chambless L., He M., Folsom A.R., Mosley T., Boerwinkle E., Ballantyne C.M. (2012). Common carotid artery intima-media thickness is as good as carotid intima-media thickness of all carotid artery segments in improving prediction of coronary heart disease risk in the Atherosclerosis Risk in Communities (ARIC) study. Eur. Heart J..

[36] Den Ouden M.E., Schuurmans M.J., Arts I.E., Grobbee D.E., Bots M.L., van den Beld A.W., Lamberts S.W., van der Schouw Y.T. (2013). Atherosclerosis and physical functioning in older men, a longitudinal study. J. Nutr. Health Aging.

[37] Wikstrand J. (2007). Methodological considerations of ultrasound measurement of carotid artery intima-media thickness and lumen diameter. Clin. Physiol. Funct. Imaging.

[38] Kanters S.D., Algra A., van Leeuwen M.S., Banga J.D. (1997). Reproducibility of in vivo carotid intima-media thickness measurements: A review. Stroke.

[39] Cooper K.D., Shafer A.B. (2019). Validity and Reliability of the Polar A300’s Fitness Test Feature to Predict VO2max. Int. J. Exerc. Sci..

[40] Cook G. (2003). Athletic Body in Balance.

[41] Aliosaitiene U., Petrulioniene Z., Rinkūnienė E., Mainelis A., Barysiene J., Smailyte U., Sileikiene V., Laucevicius A. (2024). Early Atherosclerosis in Familial Hypercholesterolemia Patients: Significance of Vascular Markers for Risk Stratification. J. Cardiovasc. Dev. Dis..

[42] Haynes W., Dubitzky W., Wolkenhauer O., Cho K.-H., Yokota H. (2013). Bonferroni Correction. Encyclopedia of Systems Biology.

[43] Sun P., Liu L., Liu C., Zhang Y., Yang Y., Qin X., Li J., Cao J., Zhang Y., Zhou Z. (2020). Carotid Intima-Media Thickness and the Risk of First Stroke in Patients with Hypertension. Stroke.

[44] Allan P.L., Mowbray P.I., Lee A.J., Fowkes F.G. (1997). Relationship between carotid intima-media thickness and symptomatic and asymptomatic peripheral arterial disease. The Edinburgh Artery Study. Stroke.

[45] Di Bello V., Carerj S., Perticone F., Benedetto F., Palombo C., Talini E., Giannini D., La Carrubba S., Antonini-Canterin F., Di Salvo G. (2009). Carotid intima-media thickness in asymptomatic patients with arterial hypertension without clinical cardiovascular disease: Relation with left ventricular geometry and mass and coexisting risk factors. Angiology.

[46] Ferreira J., Girerd N., Bozec E., Machu J., Boivin J., London G., Zannad F., Rossignol P. (2016). Intima-Media Thickness Is Linearly and Continuously Associated with Systolic Blood Pressure in a Population-Based Cohort (STANISLAS Cohort Study). J. Am. Heart Assoc..

[47] Kannel W.B. (2000). Risk stratification in hypertension: New insights from the Framingham Study. Am. J. Hypertens..

[48] Massimo P., Paolo P., Marco Z., Francesca D., Carmen T., Marcello R., Paolo P. (2008). Increase in Carotid Intima-Media Thickness in Grade I Hypertensive Subjects. Hypertension.

[49] Williams K.J., Tabas I. (1995). The response-to-retention hypothesis of early atherogenesis. Arterioscler. Thromb. Vasc. Biol..

[50] Łoboz-Rudnicka M., Jaroch J., Bociąga Z., Rzyczkowska B., Uchmanowicz I., Polański J., Dudek K., Szuba A., Łoboz-Grudzień K. (2016). Impact of cardiovascular risk factors on carotid intima-media thickness: Sex differences. Clin. Interv. Aging.

[51] Fryar C.D., Kruszon-Moran D., Gu Q., Ogden C.L. (2018). Mean Body Weight, Height, Waist Circumference, and Body Mass Index Among Adults: United States, 1999–2000 through 2015–2016.

[52] Hales C., Carroll M., Fryar C., Ogden C. (2020). Prevalence of Obesity and Severe Obesity Among Adults: United States, 2017–2018.

[53] Esselstyn C.B. (2017). A plant-based diet and coronary artery disease: A mandate for effective therapy. J. Geriatr. Cardiol..

[54] Franco O.H., de Laet C., Peeters A., Jonker J., Mackenbach J., Nusselder W. (2005). Effects of physical activity on life expectancy with cardiovascular disease. Arch. Intern. Med..

[55] Khan S.S., Ning H., Wilkins J.T., Allen N., Carnethon M., Berry J.D., Sweis R.N., Lloyd-Jones D.M. (2018). Association of Body Mass Index with Lifetime Risk of Cardiovascular Disease and Compression of Morbidity. J. Am. Med. Assoc. Cardiol..

[56] Ma H., Wang X., Xue Q., Li X., Liang Z., Heianza Y., Franco O.H., Qi L. (2023). Cardiovascular Health and Life Expectancy Among Adults in the United States. Circulation.

[57] Taekema D.G., Gussekloo J., Maier A.B., Westendorp R.G., de Craen A.J. (2010). Handgrip strength as a predictor of functional, psychological and social health. A prospective population-based study among the oldest old. Age Ageing.

[58] Bohannon R.W. (2015). Muscle strength: Clinical and prognostic value of hand-grip dynamometry. Curr. Opin. Clin. Nutr. Metab. Care.

[59] Byrkjeland R., Stensæth K.H., Anderssen S., Njerve I.U., Arnesen H., Seljeflot I., Solheim S. (2016). Effects of exercise training on carotid intima-media thickness in patients with type 2 diabetes and coronary artery disease. Influence of carotid plaques. Cardiovasc. Diabetol..

[60] Dvoretskiy S., Lieblein-Boff J.C., Jonnalagadda S., Atherton P.J., Phillips B.E., Pereira S.L. (2020). Exploring the Association between Vascular Dysfunction and Skeletal Muscle Mass, Strength and Function in Healthy Adults: A Systematic Review. Nutrients.

[61] Sixt S., Beer S., Blüher M., Korff N., Peschel T., Sonnabend M., Teupser D., Thiery J., Adams V., Schuler G. (2010). Long- but not short-term multifactorial intervention with focus on exercise training improves coronary endothelial dysfunction in diabetes mellitus type 2 and coronary artery disease. Eur. Heart J..

[62] Ibanez B., Garcia-Lunar I., Fuster V. (2022). The Intima-Media Thickness Age Is Over: The Time of Multiterritorial Subclinical Plaque Quantification Has Come. J. Am. Coll. Cardiol..

[63] Berry J.D., Willis B., Gupta S., Barlow C.E., Lakoski S.G., Khera A., Rohatgi A., de Lemos J.A., Haskell W., Lloyd-Jones D.M. (2011). Lifetime risks for cardiovascular disease mortality by cardiorespiratory fitness levels measured at ages 45, 55, and 65 years in men. The Cooper Center Longitudinal Study. J. Am. Coll. Cardiol..

[64] Gando Y., Yamamoto K., Kawano H., Murakami H., Ohmori Y., Kawakami R., Sanada K., Higuchi M., Tabata I., Miyachi M. (2011). Attenuated age-related carotid arterial remodeling in adults with a high level of cardiorespiratory fitness. J. Atheroscler. Thromb..

