# Supplementary material for: Associations between Multiple Health Indicators and Carotid Artery Intima-Media Thickness in A Healthy and Active Elderly Population
Source: J Cardiovasc Dev Dis. 2024 Mar 28;11(4):101. doi: 10.3390/jcdd11040101 (PMC11050605; doi:10.3390/jcdd11040101)
Supplement: Supplementary file 1 [file jcdd-11-00101-s001.zip › jcdd-2915931-supplementary.pdf]

Supplementary material

Supplementary Table S1. MULTIPLE COMPARISON BONFERRONI

Dependent Variable	CIMT categories	CIMT categories	Mean Difference	Std. Error	Sig.	95% Confidence Interval	
						Lower Bound	Upper Bound
Age	Low	Middle	-2.91	0.49	<.001	-4.07	-1.75
		High	-6.70	0.61	<.001	-8.16	-5.23
	Middle	Low	2.91	0.49	<.001	1.75	4.07
		High	-3.79	0.52	<.001	-5.03	-2.55
	High	Low	6.70	0.61	<.001	5.23	8.16
		Middle	3.79	0.52	<.001	2.55	5.03
Systolic	Low	Middle	-5.62	1.27	<.001	-8.67	-2.56
		High	-11.47	1.62	<.001	-15.35	-7.60
	Middle	Low	5.62	1.27	<.001	2.56	8.67
		High	-5.86	1.36	<.001	-9.13	-2.59
	High	Low	11.47	1.62	<.001	7.60	15.35
		Middle	5.86	1.36	<.001	2.59	9.13
Diastolic	Low	Middle	-0.97	0.75	0.59	-2.77	0.83
		High	-2.65	0.95	0.02	-4.94	-0.37
	Middle	Low	0.97	0.75	0.59	-0.83	2.77
		High	-1.69	0.80	0.11	-3.61	0.24
	High	Low	2.65	0.95	0.02	0.37	4.94
		Middle	1.69	0.80	0.11	-0.24	3.61
Weight (pounds)	Low	Middle	-3.08	2.63	0.73	-9.40	3.23
		High	-16.67	3.52	<.001	-25.10	-8.23
	Middle	Low	3.08	2.63	0.73	-3.23	9.40
		High	-13.58	3.04	<.001	-20.87	-6.30
	High	Low	16.67	3.52	<.001	8.23	25.10
		Middle	13.58	3.04	<.001	6.30	20.87
Height (inches)	Low	Middle	-0.62	0.31	0.14	-1.36	0.12
		High	-1.98	0.41	<.001	-2.97	-1.00
	Middle	Low	0.62	0.31	0.14	-0.12	1.36
		High	-1.37	0.35	<.001	-2.22	-0.52
	High	Low	1.98	0.41	<.001	1.00	2.97
		Middle	1.37	0.35	<.001	0.52	2.22
BMI	Low	Middle	-0.01	0.32	1.00	-0.78	0.77
		High	-1.03	0.43	0.05	-2.06	0.01
	Middle	Low	0.01	0.32	1.00	-0.77	0.78
		High	-1.02	0.37	0.02	-1.91	-0.12
	High	Low	1.03	0.43	0.05	-0.01	2.06
		Middle	1.02	0.37	0.02	0.12	1.91
Functional strength	Low	Middle	-0.62	0.26	0.05	-1.24	-0.01
		High	0.20	0.34	1.00	-0.62	1.01
	Middle	Low	0.62	0.26	0.05	0.01	1.24
		High	0.82	0.29	0.02	0.12	1.52
	High	Low	-0.20	0.34	1.00	-1.01	0.62
		Middle	-0.82	0.29	0.02	-1.52	-0.12
	Low	Middle	-3.34	0.94	0.00	-5.60	-1.08

Handgrip Left (kg)		High	-3.71	1.18	0.01	-6.55	-0.87
	Middle	Low	3.34	0.94	0.00	1.08	5.60
		High	-0.37	1.00	1.00	-2.75	2.02
	High	Low	3.71	1.18	0.01	0.87	6.55
		Middle	0.37	1.00	1.00	-2.02	2.75
Handgrip Right (kg)	Low	Middle	-2.05	0.96	0.10	-4.36	0.25
		High	-2.73	1.21	0.07	-5.63	0.17
	Middle	Low	2.05	0.96	0.10	-0.25	4.36
		High	-0.68	1.02	1.00	-3.11	1.76
	High	Low	2.73	1.21	0.07	-0.17	5.63
		Middle	0.68	1.02	1.00	-1.76	3.11
Predicted VO_2 max	Low	Middle	-0.26	0.84	1.00	-2.27	1.76
		High	0.78	1.11	1.00	-1.88	3.44
	Middle	Low	0.26	0.84	1.00	-1.76	2.27
		High	1.04	0.98	0.87	-1.31	3.38
	High	Low	-0.78	1.11	1.00	-3.44	1.88
		Middle	-1.04	0.98	0.87	-3.38	1.31

Supplementary Table S2. CORRELATIONS WITHOUT CONTROLS

	Age [n=1818]	Systolic BP [n=1046]	Dias - BP [n=1045]	Weight [n=975]	Height [n=978]	BMI [n=974]	Functional Strength [n=467]	Handgrip [n=871]	Handgrip R [n=871]	Predicted VO_2 max [n=611]
R	0.283	0.253	0.074	0.17	0.153	0.07	-0.025	0.132	0.074	-0.035
P	<0.001	<0.001	0.016	<0.001	<0.001	0.029	0.597	<0.001	0.029	0.382
